# Spatial familial networks to infer demographic structure of wild populations

**DOI:** 10.1002/ece3.7345

**Published:** 2021-03-17

**Authors:** Samantha McFarlane, Micheline Manseau, Paul J. Wilson

**Affiliations:** ^1^ Environmental and Life Sciences Department Trent University Peterborough ON Canada; ^2^ Landscape Science and Technology Division Environment and Climate Change Canada Ottawa ON Canada

**Keywords:** boreal caribou, dispersal, familial network, fitness, network analysis, pedigree reconstruction, *Rangifer tarandus*

## Abstract

1. In social species, reproductive success and rates of dispersal vary among individuals resulting in spatially structured populations. Network analyses of familial relationships may provide insights on how these parameters influence population‐level demographic patterns. These methods, however, have rarely been applied to genetically derived pedigree data from wild populations.
2. Here, we use parent–offspring relationships to construct familial networks from polygamous boreal woodland caribou (*Rangifer tarandus caribou*) in Saskatchewan, Canada, to inform recovery efforts. We collected samples from 933 individuals at 15 variable microsatellite loci along with caribou‐specific primers for sex identification. Using network measures, we assess the contribution of individual caribou to the population with several centrality measures and then determine which measures are best suited to inform on the population demographic structure. We investigate the centrality of individuals from eighteen different local areas, along with the entire population.
3. We found substantial differences in centrality of individuals in different local areas, that in turn contributed differently to the full network, highlighting the importance of analyzing networks at different scales. The full network revealed that boreal caribou in Saskatchewan form a complex, interconnected familial network, as the removal of edges with high betweenness did not result in distinct subgroups. Alpha, betweenness, and eccentricity centrality were the most informative measures to characterize the population demographic structure and for spatially identifying areas of highest fitness levels and family cohesion across the range. We found varied levels of dispersal, fitness, and cohesion in family groups.
4. *Synthesis and applications*: Our results demonstrate the value of different network measures in assessing genetically derived familial networks. The spatial application of the familial networks identified individuals presenting different fitness levels, short‐ and long‐distance dispersing ability across the range in support of population monitoring and recovery efforts.

## Graphical abstract

Network analyses of familial relationships may provide insights on how fitness influences population‐level demographic patterns in socially structured populations. Here, we use networks to reconstruct parent‐offspring relationships and create a familial network from polygamous boreal woodland caribou (*Rangifer tarandus caribou*), assessing the contribution of individual caribou to the population with several centrality metrics and determine which metrics are best suited to inform on population demographic structure. The familial network revealed that boreal caribou in Saskatchewan form a complex, interconnected social network, and identified areas of higher fitness levels and social cohesion across the range in support of population monitoring and recovery efforts.

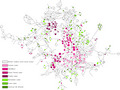

## 1 INTRODUCTION

Population genetic analyses are used to inform on the genetic composition of a population and the forces that explain the changes to that composition (Griffiths et al., 2000). A larger number of analytical approaches have been developed to delineate populations and assess the extent and patterns of gene flow and dispersal (e.g., Galpern et al., 2014; Jombart et al., 2008; Pritchard, Stephens, & Donnelly, 2000). More recently, graph‐theoretic approach has been used to assess population genetic structure (Dyer & Nason, 2004), investigate sex‐specific dispersal processes and network structures in landscape genetics (Bertrand et al., 2017), and analyze spatial patterns of genetic variation across a species’ range (Fortuna et al., 2009). In parallel, pedigree reconstructions have been done to inform on demographic parameters (Creel et al., 2003; Gobush et al., 2009; Lucena‐Perez et al., 2018; McFarlane et al., 2018), yet network analyses and genetically derived pedigrees have been used as two separate methodological frameworks. Here, we suggest that the combination of these methods may highlight the interconnectedness between individuals (Escoda et al., 2019; Morrison, 2016), differences in reproductive success (McFarlane et al., 2018), and ultimately inform on the demographic structure of a population.

Reconstructing a reasonably complete and accurate familial network from pedigree data is especially relevant for endangered species, providing information on mating patterns and reproductive success (Lucena‐Perez et al., 2018; Manlik et al., 2016). However, collecting reliable parentage information for cryptic and elusive species is difficult or directly unfeasible; pedigree information obtained through direct field observations is often limited to females and may consistently overlook cryptic mating (Coltman et al., 1999; Gottelli et al., 2007). Molecular markers, such as microsatellites, have been used to infer parentage and familial relationships in wild populations (Pemberton, 2008) and assess individual heterogeneity in survival and reproduction (Bolnick et al., 2011; Hamel et al., 2009; Kendall et al., 2011). Such heterogeneity can be the result of a number of common processes, such as persistent social rank (e.g., von Holst et al., 2002; Stockley & Bro‐Jørgensen, 2011), unequal allocation during parental care (e.g., Johnstone, 2004; Manser & Avey, 2000), fine‐scale spatial habitat heterogeneity (Bollinger & Gavin, 2004; Franklin et al., 2000; Manolis et al., 2002), and genetics (Meyers & Bull, 2002; Nussey, 2005).

Graph theory (Harary, 1969) is widely used in ecology to assess functional and structural connectivity (Fall et al., 2007; Urban & Keitt, 2001; Wagner & Fortin, 2005). Graphs are represented as a network of nodes and edges, where edges imply a level of connection between the nodes (Urban & Keitt, 2001). Several network‐based measures are commonly used to quantify indirect connections between nodes (e.g., individuals, habitat patches; Table 1). Each measure captures a distinct aspect of the network. Alpha centrality is a generalization of eigenvector centrality given to directed graphs; while eigenvector centrality is a measure of the influence of a node in a network, alpha centrality allows nodes to have external sources of influence that does not depend on that node's connection to other nodes (Bonacich & Lloyd, 2001). Betweenness centrality indicates how central a node is in a network, based on the number of shortest paths between pairs of nodes that pass through that node (Freeman, 1977). Closeness centrality measures how fast information can spread from a given node to all other reachable nodes in a network, and the Latora closeness centrality is used in networks with disconnected components (Latora and Marchiori, 2001). Degree centrality represents the number of edges connected to a node; in directed graphs, in‐degree counts the number of edges directed toward the node, and out‐degree counts the number of edges that leaves the node toward other nodes (Harary, 1969). Eccentricity centrality is the maximum distance from a node to any other node, representing the importance of a node within a network, determining the influence of a particular node within a network (Hage & Harary, 1995). A priori selection of network measures is important to avoid including several spuriously correlated measures (Webber et al., 2020). Although some network‐based centrality measures may overlap, each measure captures a distinct aspect of the network; nodes with high scores for one measure may not necessarily have a high score in other measures.

**TABLE 1:** Node‐based measures of connectivity

Metric	Type	Definition
Alpha centrality	Indirect	Alpha centrality of all vertices. A generalization of eigenvector centrality to directed graphs. Alpha centrality indicates the overall connectivity of a node, both direct and indirect connections (Bonanich and Lloyd 2001).
Betweenness centrality	Indirect	Quantifies the number of times a node lies along the shortest path between two other nodes in the network (Freeman, 1977).
Closeness centrality	Indirect	A centrality measure based on the shortest path length between a node and other nodes in the network. The Latora closeness centrality is used in networks with disconnected components (Latora and Marchiori 2001).
Degree centrality	Direct	The number of edges connected to a node (Harary, 1969).
Eccentricity centrality	Indirect	The maximum noninfinite length of a shortest path between *n* and another node in the network (Hage & Harary, 1995).

Here, we infer population demographic structure by assessing different node‐based measures of centrality obtained from a familial pedigree network. First, we use microsatellite data to identify parent–offspring relationships and construct a spatial familial network from all relationships (familial pedigree) of boreal caribou in Saskatchewan, Canada. Then, we create a spatial familial network to identify local area networks with varying distributions of centrality measures, determining whether high centrality measures and edge‐to‐node ratios at the fine scale correspond to high centrality in the full network. Spatially analyzing familial networks is inherently difficult due to the presence of inferred individuals, whose spatial locations are unknown. By using the centrality measures from the aspatial network in the spatial network of individuals, the network connections to the inferred individuals can be brought into a spatial framework. We also assess the structure and cohesiveness within the full network using edge removal to identify boundaries that run between subgroups (Girvan & Newman, 2002; Lusseau & Newman, 2004; Newman & Girvan, 2004), with a particular focus on parts of the range presenting different levels of anthropogenic disturbance. Our findings allow us to discuss how different measures of network centrality can be used to spatially identify areas of highest fitness levels, dispersal and reproductive skew across the landscape in support of population monitoring and recovery efforts.

## 2 MATERIALS AND METHODS

Boreal caribou are part of the Boreal Caribou designatable unit (COSEWIC, 2011), listed as Threatened under the federal *Species at Risk Act* (Environment Canada, 2012) and as Vulnerable in Saskatchewan (SKCDC, 2020). In response to the listing, the Government of Saskatchewan initiated a comprehensive monitoring program along with range planning efforts with the goal of achieving a self‐sustaining boreal caribou population (Johnson et al., 2020; Saskatchewan Ministry of Environment, 2013). The southern range boundary of boreal caribou in Saskatchewan has moved northward over the last century, and habitat in the Boreal Plains has become increasingly fragmented and reduced in area (Arsenault, 2003; Rock, 1992). Further studies have shown reduced movement of female caribou and low adult survival in the Boreal Plains (Arsenault & Manseau, 2011). Boreal caribou in Saskatchewan maintain a natural clinal pattern of genetic structure, with isolation by distance and isolation by resistance shaping spatial patterns of genetic variation (Galpern et al., 2012; Galpern Manseau & Wilson, 2012; Priadka et al., 2018). More information on Saskatchewan's boreal caribou habitat can be found in Appendix 1.

### 2.1 Fecal pellet collection and genetic analysis

We used samples from across the boreal caribou range in Saskatchewan, Canada, collected during winters of 2013–2019 (Figure S1.1; Table 2). This dataset was assembled primarily from systematic noninvasive fecal pellet surveys where aerial transects were systematically flown using a fixed‐wing aircraft to locate caribou catering locations (sites where caribou paw to uncover terrestrial lichens). Additional samples (90) from the northern part of the Saskatchewan Boreal Shield were obtained from blood blots or vials collected from individual boreal caribou handled during radio‐collaring (McLoughlin et al., 2019; Priadka et al., 2018). All samples were kept frozen at −20°C until DNA extraction was performed.

**TABLE 2:** Sampling data

Sampling area	Survey years	Sample type	Number of samples collected	Number of samples successfully scored	Number of unique genotypes	Genotyping success (%)	Dropouts (%)	False alleles (%)
Flin Flon	2014	Fecal	336	320	104	95.2	0.0077	0.032
La Ronge	2013 and 2015	Fecal	497	403	162	81.1	0.0032	0.0097
SK Boreal Plains West	2016	Fecal	242	233	122	96.3	0	0
Patterson Lake	2018	Fecal	21	19	9	90.5	0.0089	0.036
SK2 Central	2017	Fecal	452	371	150	82.1	0	0
SK Shield	2014	Fecal	99	98	98	99	0	0
SK Shield	2019	Blood	551	526	288	95.5	0	0
Total	—	—	2,198	1,970	933	—	—	—

In order to generate individual‐specific genetic profiles and familial pedigree networks, DNA samples were amplified at 15 variable microsatellite loci (BM848, BM888, Map2C, Bishop et al. (1994); FCB193, Buchanan and Crawford (1993); NVHRT16, Røed and Midthjell (1998); OHEQ, Jones et al. (2000); RT1, RT5, RT6, RT7, RT9, RT13, RT24, RT27, RT30, Wilson et al. (1997)) along with caribou‐specific Zfx/Zfy primers for sex identification. DNA was extracted by removing the mucosal layer of cells coating the fecal pellets and followed the extraction protocol outlined in Ball et al. (2007). Microsatellite alleles were scored with the program GeneMarker® (SoftGenetics, State College, PA) and followed a protocol documented in Flasko et al. (2017). Unique individuals were identified using the program ALLELEMATCH (Galpern, Manseau, Hettinga, et al., 2012). We retained samples that amplified at ≥ 5 loci and re‐amplified apparent unique genetic profiles represented by a single sample using two independent scorers to confirm unique individual identities (Hettinga et al., 2012). The rate of allelic dropouts (amplifications of only one of the two alleles for heterozygous individuals, producing false homozygotes; Taberlet et al. 1996) and false alleles (false allele amplifications; Bonin et al. 2004) were calculated using these re‐amplification results.

### 2.2 Defining familial relationships between individuals

We identified familial relationships of boreal caribou in the study area by reconstructing parent–offspring relationships using COLONY v2.0.6.5 (Jones & Wang, 2010). We calculated population allele frequencies using GenAlEx v6.5 (Peakall & Smouse, 2012). Input parameters were set to allow for female and male polygynous mating systems without inbreeding avoidance, and the probability of mothers or fathers being present in the sampled dataset was set to 50% in the absence of other prior information. All sampled females were set as possible mothers, and all sampled males were set as possible fathers. COLONY infers the parental genotypes for each individual; inferred parents are genotypes that are not included in the candidate parent samples, either through that individual's genotype not being captured during sampling, or that parent is no longer living, resulting in a family network with more individuals than were sampled. Finally, individual fitness was calculated with the number of offspring each individual produced.

### 2.3 Modeling the demographic structure of the population

Identifying parts of the network that are highly connected and those individuals that are less connected to the network can help define the local and global structure of the familial network. We used the r package *CINNA* (Ashtiani et al., 2018) to calculate individual node‐based measures of network centrality. Nodes represent individuals, and edges represent parent–offspring relationships, with directionality from parent to offspring. We calculated five direct and indirect node‐based measures of centrality for each individual to quantify distinct aspects of centrality: alpha, betweenness, closeness, degree, and eccentricity centrality (Table 1). We calculated correlation coefficients between measures to only select statistically independent aspects of centrality. We used principal component analysis (PCA) to collapse variance among any dependent centrality measures, as suggested by Brent (2015), and to identify the most important centrality types based on our network structure. We used the r package *FactoMineR* (Lê et al., 2008) to run the PCA, and package *factoextra* (Kassambara & Mundt, 2020) to visualize PCA results.

#### 2.3.1 Network analysis

As boreal caribou mating system is polygamous, with individuals having multiple mating partners, a dense and complicated network is created; visually analyzing the aspatial network along with the node‐based measures of network centrality allows for easier identification of patterns and trends within the network. We used Cytoscape v3.7.2 (Shannon et al., 2003) for the nonspatial analyses of the local and full familial networks. We created the familial network from the reconstructed parent–offspring relationships identified by COLONY. As each individual has their parents identified by COLONY, as well their offspring, a network can be created from the multigenerational relationships among individuals.

To assess network cohesiveness within the full network, we used the Girvan–Newman algorithm to look for boundaries that run between family groups to find natural divisions within the network by removing edges with the highest betweenness scores (Girvan & Newman, 2002; Lusseau & Newman, 2004; Newman & Girvan, 2004). We used an edge betweenness centrality measure (Freeman, 1977) calculated in the NetworkAnalyzer (Assenov et al., 2007) plugin for Cytoscape. Edge betweenness quantifies how often an edge is crossed when moving between any pair of individuals in the network; bottlenecks are identified in edges that have higher betweenness, as these edges are passed the most often when connecting individuals. Edges were systematically removed until groups can be identified.

#### 2.3.2 Spatial application of network analysis

We examined how local areas presenting high and low edge‐to‐node ratios (Box 1) contributed to the full network by comparing centrality measures across local areas within the network. The local areas were of management interest, had a comparable number of individuals and similar geographic sizes. We plotted the spatial locations of all sampled individuals and parent–offspring relationships in ArcGIS (ESRI Inc., 2018) to spatially identify local areas. Local areas were defined based on visual inspection of the sample locations, where areas with a large number of samples identified as local areas, and from these, we selected areas with the highest and lowest ratio of edges (parent‐offspring relationships) to nodes (individuals) within the same local area to compare local area networks within the larger spatial familial network. Identifying local areas with a high number of edge‐to‐node ratios highlights areas within the full network presenting different degrees of familial cohesion, or where parent–offspring remain in the same geographical area. We examined the centrality measures for all sampled individuals within each local area network, as well as for their first neighbors (individuals one degree away from individuals in these areas—as inferred parents do not have spatial locations, this captures inferred individuals) and compared each local area network.

BOX 1 **Edge‐to‐node ratio definition for local areas. Arrows indicate the direction of parent–offspring relationships. Edge‐to‐node ratio calculated by dividing number of edges within the local area by the number of individuals within the local area.**

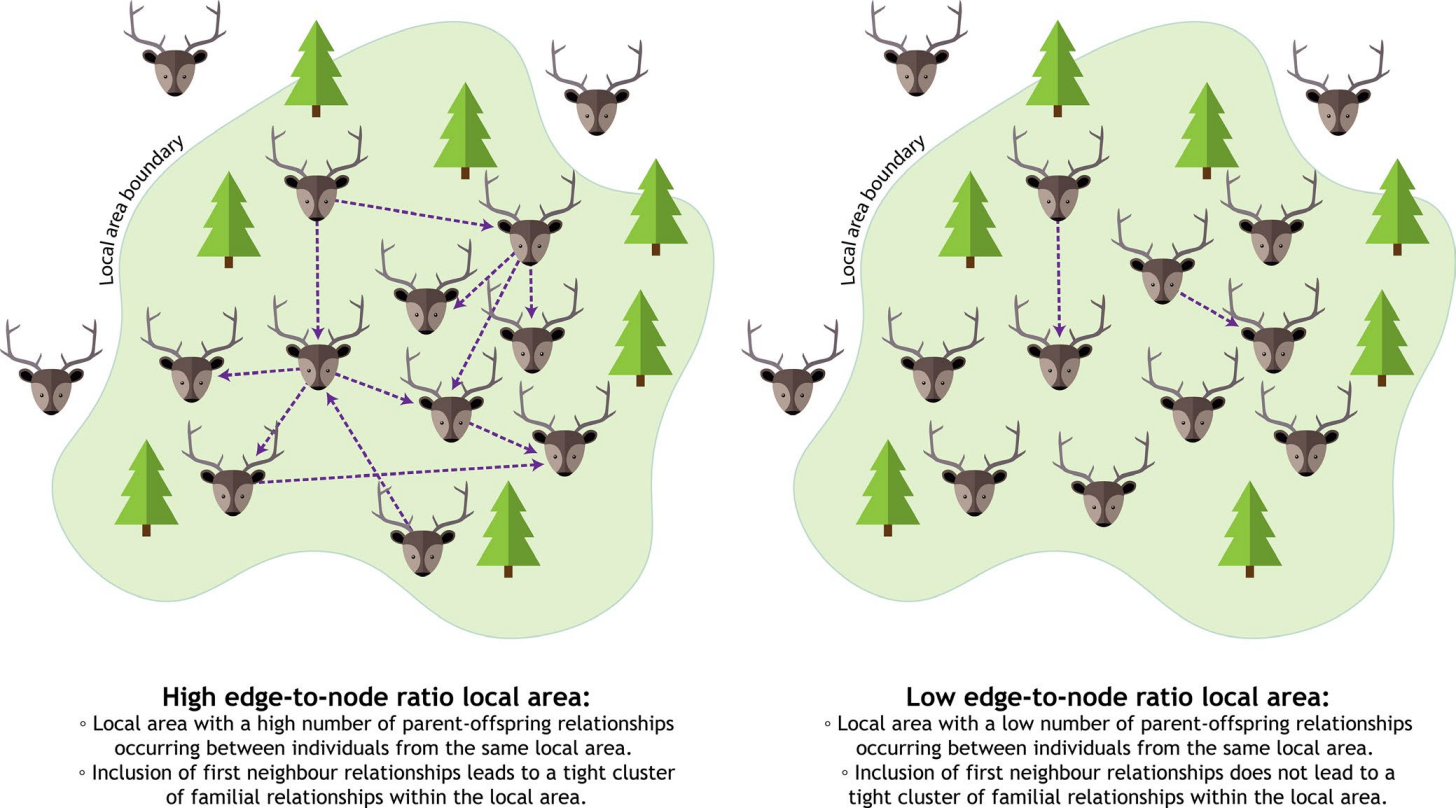

## 3 RESULTS

**FIGURE 1:**
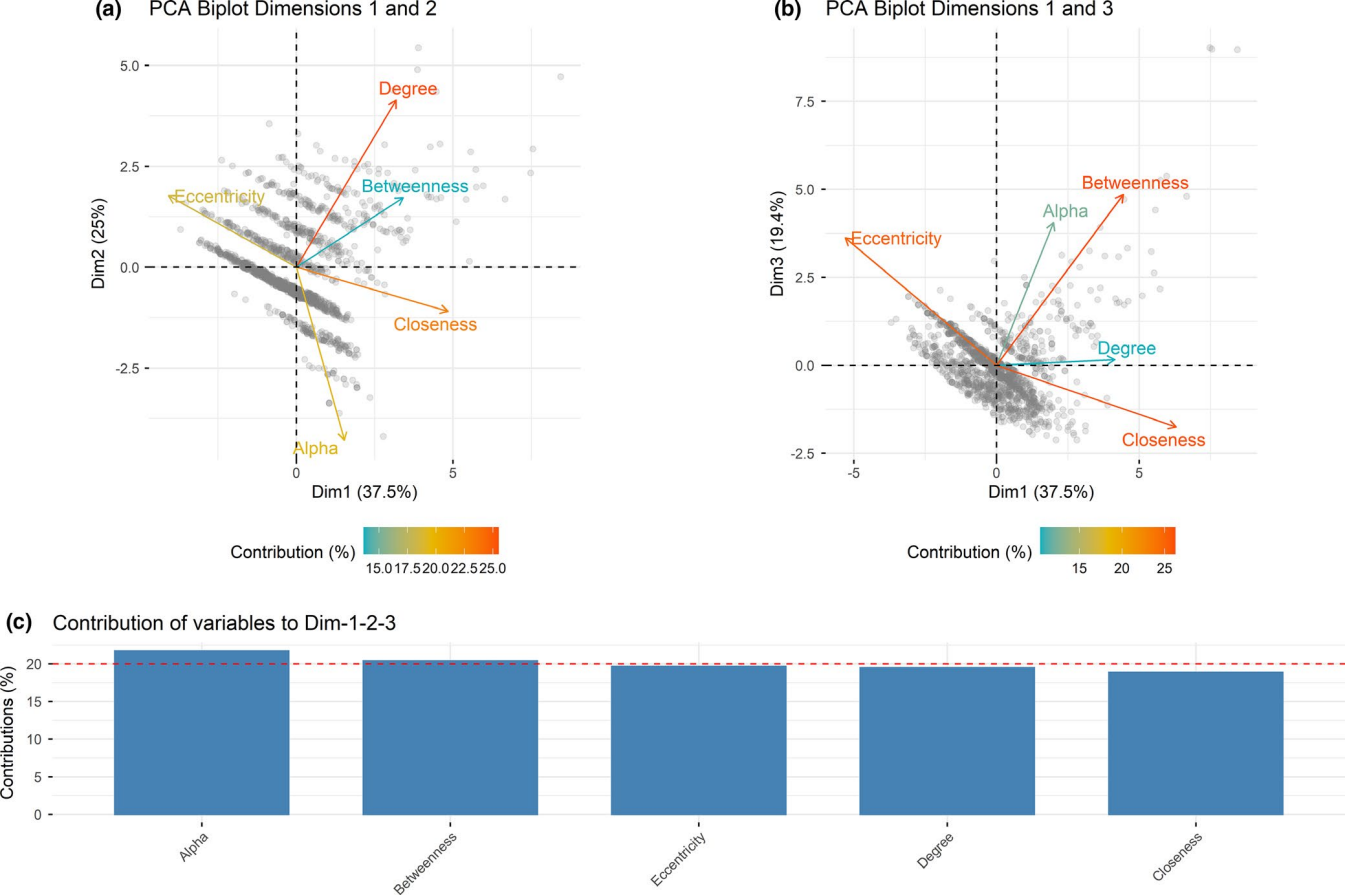
Principal component analysis (PCA) results for the node‐based centrality measures. (a) PCA results for PC1 and PC2; (b) PCA results for PC1 and PC3; (c) contributions of node‐based centrality measures in accounting for variability in PCs 1–3. The red dashed line represents the expected average contribution

A total of 2,198 samples were collected (2,099 fecal and 99 blood blot). 1,970 were successfully scored (average success rate of 91.4%), and 933 unique individuals were identified (Table 2), representing roughly 20% of the estimated population abundance in Saskatchewan (S. McFarlane, unpublished data). Overall, the average dropout rate was 0.0028% and the average false allele rate was 0.011%. Pedigree reconstruction inferred an additional 310 females and 319 males, for a total familial network of 1,562 individuals. 355 males and 360 females were identified as parents. 1,487 (95.2%) individuals were linked in one network, with the remaining 75 individuals linked in five smaller clusters (Figure S2.1). We used the 1,487 individuals identified in the primary network for calculating node‐based measures of centrality. The PCA identified alpha, betweenness, and eccentricity centrality as the centrality measures contributing the most to the components, and were all informative measures, capturing different aspects of individual centrality (Figure 1; Table 3).

**TABLE 3:** Correlation coefficients between node‐based measures of network connectivity

	Degree centrality	Eccentricity centrality	Betweenness centrality	Closeness centrality
Alpha Centrality	−0.216	−0.124	0.152	0.208
Degree Centrality		−0.118	0.371	0.284
Eccentricity Centrality			−0.11	−0.544
Betweenness Centrality				0.234

### 3.1 Spatial network analysis

#### 3.1.1 Local area networks

**FIGURE 2:**
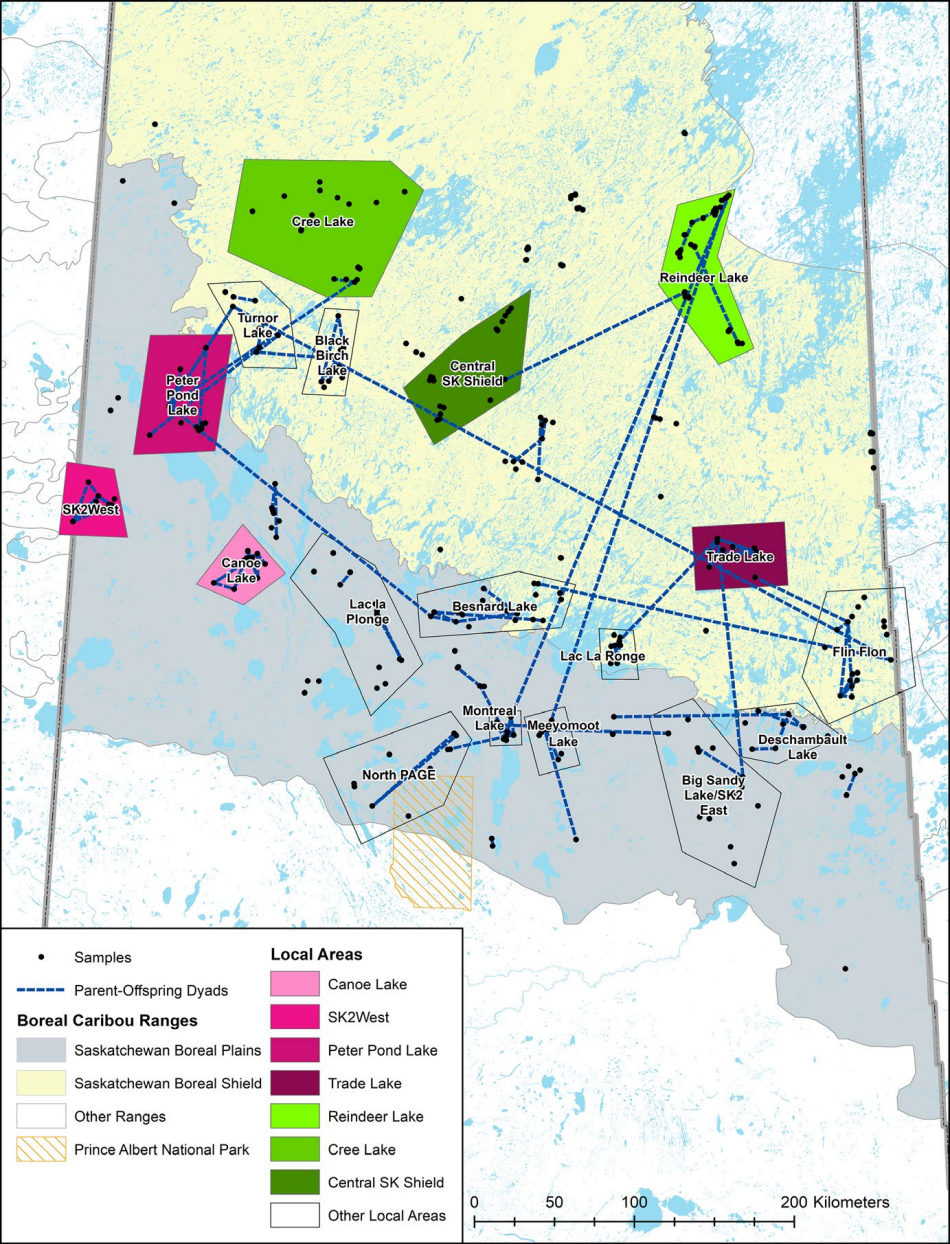
Locations of local areas. High edge‐to‐node ratio (pink) and low edge‐to‐node (green) local areas within the spatial familial network. Lines represent parent–offspring relationships

**FIGURE 3:**
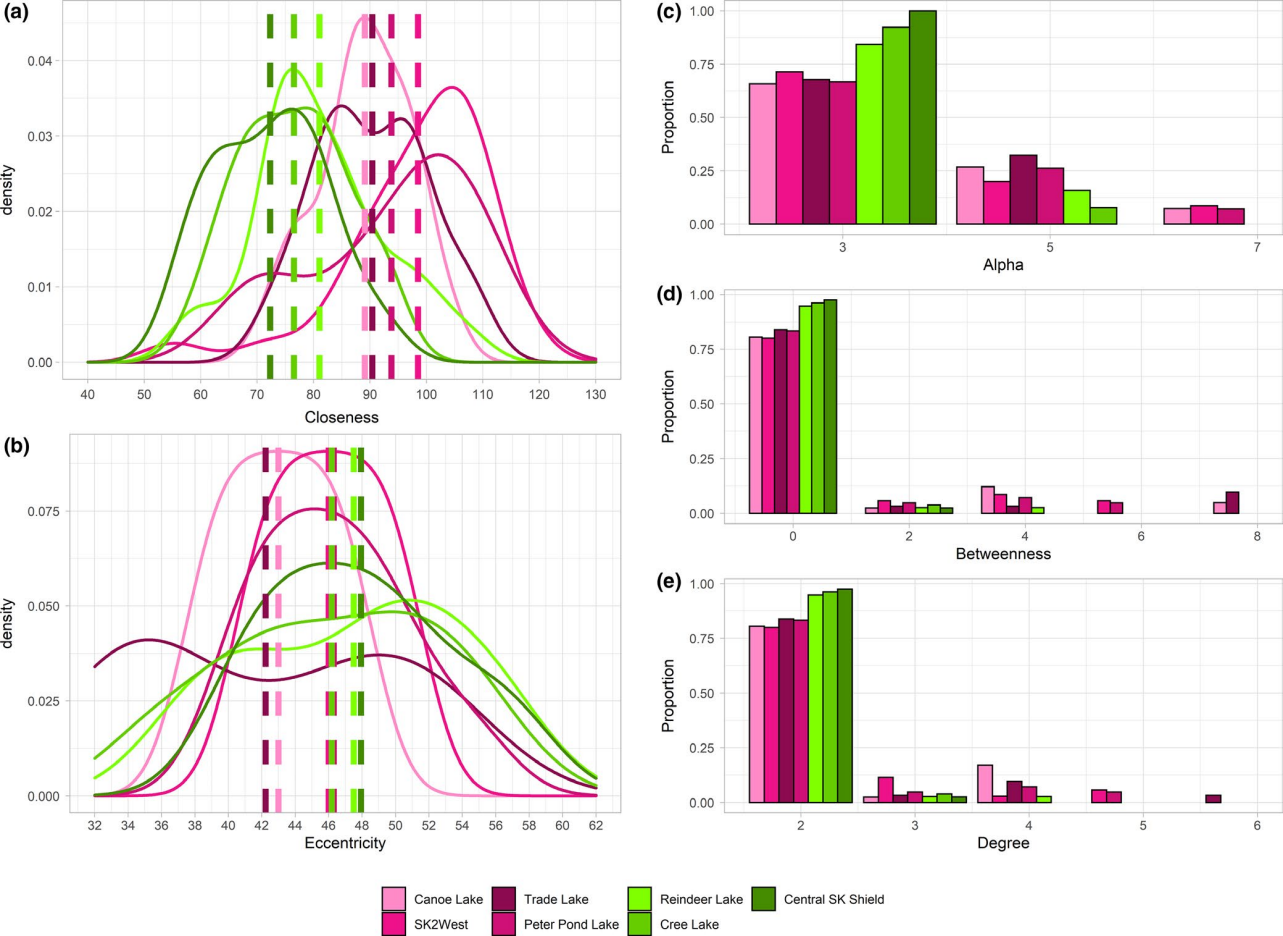
Distribution of node‐based centrality measure values for boreal caribou in high edge‐to‐node (pink) and low edge‐to‐node (green) local areas in Saskatchewan: closeness centrality (a), eccentricity centrality (b), alpha centrality (c), betweenness centrality (d), and degree centrality (e). Dashes lines in (a) and (b) represent mean centrality values

We identified 18 local area networks in order to determine the cohesiveness and centrality of individuals. The local areas with the lowest edge‐to‐node ratios were all located in the northern part of the Boreal Shield, with the high edge‐to‐node ratio areas found further south in the western part of the Boreal Plains and southern part of the Boreal Shield (Figure 2). We found differences between the distribution of centrality measures between high and low edge‐to‐node ratio local areas (Figure 3). The largest edge‐to‐node ratio was Canoe Lake in the western Boreal Plains (ratio of 15; Table S2.1, Figure S2.3). We identified three other local areas with similarly high edge‐to‐node ratios (Figure S2.4, Figure S2.5, Figure S2.6, Table S2.1). The smallest edge‐to‐node ratio (Central SK Shield) had zero parent–offspring relationships (Table S2.1; Figure S2.7). We identified two other local areas with similarly low edge‐to‐node ratios, with very few parent–offspring relationships occurring within these local areas (Figure S2.8‐Figure S2.9, Table S2.1), indicating that Boreal Shield individuals are not presenting the same proximity to related individuals as observed in the Boreal Plains. Overall, edge‐to‐node ratios correlated positively to closeness (Figure S2.2a), alpha (Figure S2.2c), betweenness (Figure S2.2d), and degree centrality (Figure S2.2e). However, edge‐to‐node ratios decreased with eccentricity centrality (Figure S2.2b), meaning areas with lower edge‐to‐node ratios were less central to the overall network.

When bringing in the first neighbors of all individuals within a local area, the high edge‐to‐node ratio areas formed a tighter cluster of individuals than in the low edge‐to‐node ratio areas. Including first neighbors in the area with the highest edge‐to‐node ratio (Canoe Lake) increased the ratio to 1.14 and connected 73.6% of individuals into one cluster (Figure S2.3). A large proportion of each high edge‐to‐node ratio local area became connected into one or two large clusters with the inclusion of first neighbors (Figure S2.4, Figure S2.5, Figure S2.6). In comparison, including first neighbors in the lowest edge‐to‐node ratio local area (Central SK Shield) increased the ratio to 0.86, but did not connect many individuals into one cluster (only 12.8% of individuals; Figure S2.7), meaning areas with higher edge‐to‐node ratios represent tighter clusters of familial relationships.

#### 3.1.2 Full network

**FIGURE 4:**
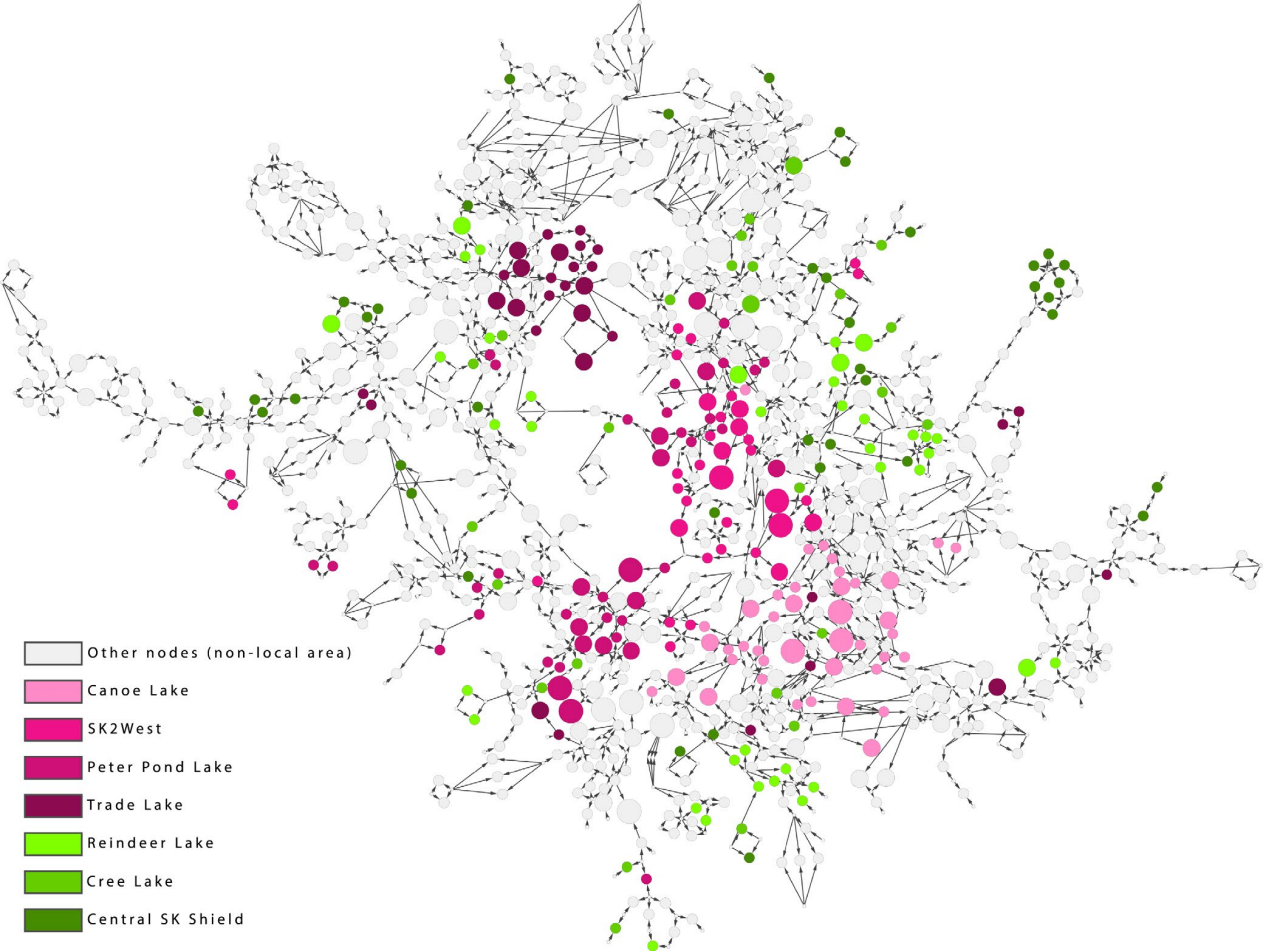
Boreal caribou familial network in Saskatchewan, Canada. Node size indicates alpha centrality score. Node colour represents both local area and edge‐to‐node ratios. All pink nodes represent individuals from local areas with high edge‐to‐node ratios, and green nodes represent individuals from local areas with low edge‐to‐node ratios

Individuals from high edge‐to‐node ratio local areas were located more centrally within the full family network and clustered with other individuals from the same local area. Individuals from low edge‐to‐node ratio local areas were dispersed throughout the network and primarily found on the outer edges of the network (Figure 4). Although all local areas were of similar geographic size (Figure 2), individuals from low edge‐to‐node ratio local areas were not closely connected to each other in the network. Individuals from these local areas were not found within a few edges of other individuals from the same local area, indicating that individuals encountered in each low edge‐to‐node ratio local area are from different familial lines, or are dispersers that were sampled in that local area (Figure 4); as the edges in the familial network represent parent–offspring relationships, these individuals are not highly related to one another and do not form a cohesive group. In contrast, individuals from high edge‐to‐node local areas were highly connected to one another within the full network, indicating they are closely related, with a high density of familial ties (parent–offspring relationships).

Removal of edges with high betweenness did not alter the overall network structure (Figure S2.10). Most edges within the network had low betweenness centrality (score of 1% – 81.5% of edges; Table 4). Only 2.97% of edges were removed after sequentially removing edges with the highest edge betweenness score until only edges with an edge betweenness > 4 remained (Table 4). While edge removal did not lead to separated subnetworks, the high edge‐to‐node local areas from the Boreal Plains remained central and clustered within the edge removal network (Figure S2.10). Individuals from Trade Lake maintained a high level of clustering, but became separated from the main network, forming a separate subgroup (Figure S2.10). Removal of high betweenness edges did not result on individuals from low edge‐to‐node ratio areas becoming separate subgroups; individuals remained dispersed throughout the network (Figure S2.10).

**TABLE 4:** Edge betweenness scores for each edge in the full familial network

Edge betweenness	Count
20	1
18	1
12	5
9	2
7	5
6	9
5	30
4	26
3	201
2	50
1	1,454

## 4 DISCUSSION

Network analyses have been used in biological and ecological studies to quantify and explore the structure of populations across numerous taxa (Bertrand et al., 2017; Dyer & Nason, 2004; Fortuna et al., 2009), but to our knowledge, this is the first to combine genetically derived pedigree data with network analysis to infer familial structure of wild populations. Network analyses are powerful and flexible methods for investigating the complex networks of interconnections between individuals within and between populations (Wasserman & Faust, 1994). With a large interconnected network of 1,562 nodes (individuals) and 1,866 edges (parent–offspring relationships) between individuals, it can be difficult to identify significant differences within the network. By bringing the familial network into a spatial framework and incorporating aspatial node‐based centrality measures, we were able to identify groups presenting different levels of cohesion within the network, with some local areas composed of clustered family groups and others presenting lower fitness or being more dispersed over the range. Comparing local area networks allowed us to identify areas of higher and lower fitness and connectivity in the overall boreal caribou familial network.

By identifying local areas within the network, we were able to gain a better understanding of which areas contributed most to the familial network. We found significant differences in centrality measures between local areas in the full familial network, and these variations in individual centrality would have remained hidden if only the full familial network was examined. We used five centrality measures in our network analysis of familial networks (Figure 3) and found that alpha, betweenness, and eccentricity centrality were the most informative measures of individual centrality (Figure 1). Degree centrality in familial networks represents the parents of an individual (in‐degree) and the offspring of an individual (out‐degree), giving a direct measure of an individual's reproductive output and fitness levels. It is important to note, however, that inferred individuals in the pedigree will always have an in‐degree of 0, as it is not possible to infer the parents of inferred individuals, and in‐degree will always be 2 for sampled individuals; in‐degree values of 1 are possible when analyzing the subgroups alone. Alpha centrality is an important metric for familial networks, as it indicates those individuals who are connected to individuals who themselves are highly connected, giving an indication of individual fitness, even if that individual does not have a lot of direct connections (offspring). Reproductive output can be highly asymmetrical, with the number of offspring varying between individuals (McFarlane et al., 2018), and alpha centrality can indicate if that individual is part of a large extended family if they are connected to highly connected individuals. McFarlane et al. (2018) found significant difference between fitness level in mountain caribou and showed that there could be genetic predisposition to higher fitness levels, with evidence of inbreeding avoidance. Maternal social rank influenced reproductive success in reindeer (*R. tarandus*), with higher fitness females having higher fecundity and earlier offspring date of birth than lower fitness females (Holand et al., 2004). We found that local areas with high edge‐to‐node ratios had a wider distribution of alpha and degree centrality, indicating that more higher fitness individuals are found in these local areas than in low edge‐to‐node local areas (Figure 2c), and are better connected to other well‐connected individuals. Three of the four high edge‐to‐node ratio local areas we identified are located in the western part of Saskatchewan's Boreal Plains, which has the highest levels of both anthropogenic and fire disturbance in the Boreal Plains (Figure S1.2), and the tight family groups we observed in these areas may be a result of decreased dispersal propensity due to high levels of fragmentation between local areas.

Betweenness centrality is another important metric for network analysis, as it captures the interconnectedness of subgroups; individuals with high betweenness interact with individuals who do not interact with one another, therefore making betweenness important for maintaining group cohesion and connecting disparate parts of the network (Brent, 2015). Our familial network was not comprised of subgroups, as most individuals (94.2%) had a betweenness centrality of 0, and 95.2% of all sampled individuals formed one large familial network. Even after the removal of edges with the highest edge betweenness, the overall network structure did not change, with most individuals still connected in one main network, with no clear subgroups (Figure S2.10). Our study species displays a polygamous mating system, with individuals potentially having multiple partners, producing a complex network of parent–offspring relationships and full‐ and half‐siblings, with high interconnectedness among individuals across the network (Figure S2.1). Our highly interconnected network with no evidence of subgroups and low average betweenness centrality is the result of the polygamous mating system and high dispersal ability.

The high eccentricity centrality and low closeness centrality inform on the presence of small numbers of closely related individuals, and generally longer distance dispersing in the Boreal Shield when compared to the Boreal Plains. The Boreal Shield is less fragmented than the Boreal Plains, with significantly less anthropogenic disturbance (Figure S1.2; Table S1.1). Very few parent–offspring relationships occurred within or between the northern Boreal Shield local areas (Figure 2). This suggests that individuals in the Boreal Shield are not central to the familial network and have lower individual fitness, not producing many offspring that survive until fall (low degree centrality). Individuals in low edge‐to‐node local areas are not from the same familial lines and are not highly related to any other individuals in the network. The removal of high betweenness edges led to some individuals becoming disconnected from the full network, but these disconnected individuals were not from one local area, instead located throughout both ecozones, again highlighting the interconnectedness of the familial network.

In most animal network studies, nodes represent observed individuals, with relationships between pairs of individuals (dyads) defined by an association index (the time the pair of individuals spent together), with edges representing observed relationships, forming an interaction network (Morrison, 2016; Whitehead & Dufault, 1999). For many species, it is not possible or feasible to directly observe rare and elusive species, and therefore, association information cannot be obtained. Pedigree reconstruction can give direct information about dyads between closely related individuals (parent–offspring and full siblings), with these relationships forming the basis of the familial network. In comparison with association networks, in familial networks, only the sampled individuals are known or observed, and the edges between individuals and the unsampled individuals (parents) are inferred by the data analysis (Morrison, 2016). Reconstructing a familial network from genetically derived pedigree data gives valuable information about the number of mating partners, the number of offspring, and the structure of the reproductive network of a population (McFarlane et al., 2018; Pemberton, 2008). Pedigrees represent historical and evolutionary connections between generations; these relationships have long been recognized as reticulating but are instead commonly presented as simplified trees instead of networks, where reticulations caused by inbreeding are absent (Morrison, 2016). Pedigrees represent a network of relationships, and therefore, reconstructed pedigrees inherently contain information that can be used to construct a network. With a wide spectrum of mating systems present in wildlife species (Clutton‐Brock, 1989), almost all species present pedigree networks, with multiple partners and/or offspring attributed to each individual, therefore creating a complex network of familial relationships (Morrison, 2016). Although caribou present varying levels of individual fitness (McFarlane et al., 2018) and their distribution is spatially clustered across the range, our network does not appear to be vulnerable to sudden population crashes resulting from changes in population structure, isolation, and inbreeding. Our network was highly connected as a result of the polygamous mating system of caribou and ability for long range dispersal. Although family groups can be identified within the network, presenting varied levels of dispersal, fitness, and cohesion, the removal of edges with high betweenness did not change the overall network structure or lead to disconnected groups. Our individual‐based familial network provides more precise information on the composition of different parts of the caribou range in Saskatchewan and their contribution to the overall population. The local areas were in some cases composed of isolated individuals presenting low fitness levels, individuals in smaller or larger groups presenting high fitness levels.

Network analyses are powerful methods to assist in wildlife conservation (Bertrand et al., 2017; Dyer & Nason, 2004; Fortuna et al., 2009), but most wild populations cannot be directly observed, and demographic networks cannot be constructed. By constructing a familial network based on genetically derived parent–offspring relationships, we calculated informative measures to draw a much finer picture of their individual fitness levels, pattern of demographic structure, and relative contribution of local areas to the larger population. The spatial application of the familial network allowed us to identify areas with individuals of higher fitness levels, short‐ and long‐distance dispersal ability across the range in support of population monitoring and recovery efforts.

## CONFLICT OF INTEREST

None declared.

## AUTHOR CONTRIBUTIONS

**Samantha McFarlane:** Conceptualization (lead); Formal analysis (lead); Investigation (lead); Methodology (lead); Software (lead); Visualization (lead); Writing‐original draft (lead); Writing‐review & editing (equal). **Micheline Manseau:** Conceptualization (equal); Data curation (equal); Formal analysis (supporting); Funding acquisition (lead); Investigation (supporting); Methodology (lead); Project administration (equal); Supervision (lead); Validation (supporting); Writing‐review & editing (lead). **Paul Wilson:** Conceptualization (supporting); Data curation (equal); Funding acquisition (lead); Project administration (lead); Resources (equal); Supervision (equal); Writing‐review & editing (equal).

### OPEN RESEARCH BADGES

This article has earned an Open Data badge for making publicly available the digitally‐shareable data necessary to reproduce the reported results. The data is available at https://doi.org/10.5061/dryad.zkh189385

## Supporting information

Supplementary Material

## Data Availability

Data deposited via the Dryad Digital Repository (https://doi.org/10.5061/dryad.zkh189385).
