# Supplementary material for: Spatial familial networks to infer demographic structure of wild populations
Source: Ecol Evol. 2021 Mar 17;11(9):4507–19. doi: 10.1002/ece3.7345 (PMC8093719; doi:10.1002/ece3.7345)
Supplement: Supplementary file 1 — Supplementary Material [file ECE3-11-4507-s001.docx]

# Appendices

## Appendix 1: Ecozone information

Saskatchewan’s Boreal Plains ecozone is characterized by low rolling forested hills and plains, interspersed with fens, bogs, marshes, and lakes (Saskatchewan Ministry of Environment, 2019). The central Boreal Plains is an important area for boreal caribou as it provides a large proportion of high-value upland (pine-lichen forest) and lowland (peat land) caribou habitat (Saskatchewan Ministry of Environment, 2019). In this area, there is a history of industrial forest management activities, which results in a network of permanent and non-permanent roads and trails (Saskatchewan Ministry of Environment, 2019). Saskatchewan’s Boreal Shield ecozone is characterized by conifer peat land complexes, muskegs and bogs, and upland moderate to dense mature conifer forests with abundance lichens (Environment Canada, 2012). Saskatchewan’s Boreal Shield represents a unique situation with very low anthropogenic disturbance and a high fire cycle, representing a relatively intact ecosystem little modified by humans, where natural ecological processes dominate (Environment Canada, 2012). Boreal caribou have disappeared from the southern edge of the Boreal Plains due to agricultural development and habitat loss linked to anthropogenic activities and are at a higher risk of loss than boreal caribou in the Boreal Shield (Saskatchewan Ministry of Environment, 2013). The proportion of area covered by anthropogenic disturbances is higher in the Boreal Plains than in the Boreal Shield (20.4% vs. 3.2%, respectively; Table S1.1), which may represent a higher source of impact on Boreal Plains’ caribou population. However, a highly active fire cycle has affected 56.5% of the Boreal Shield in the last 40 years (Table S1.1), putting Boreal Shield caribou at low to medium risk in this part of their range (Saskatchewan Ministry of Environment, 2013).


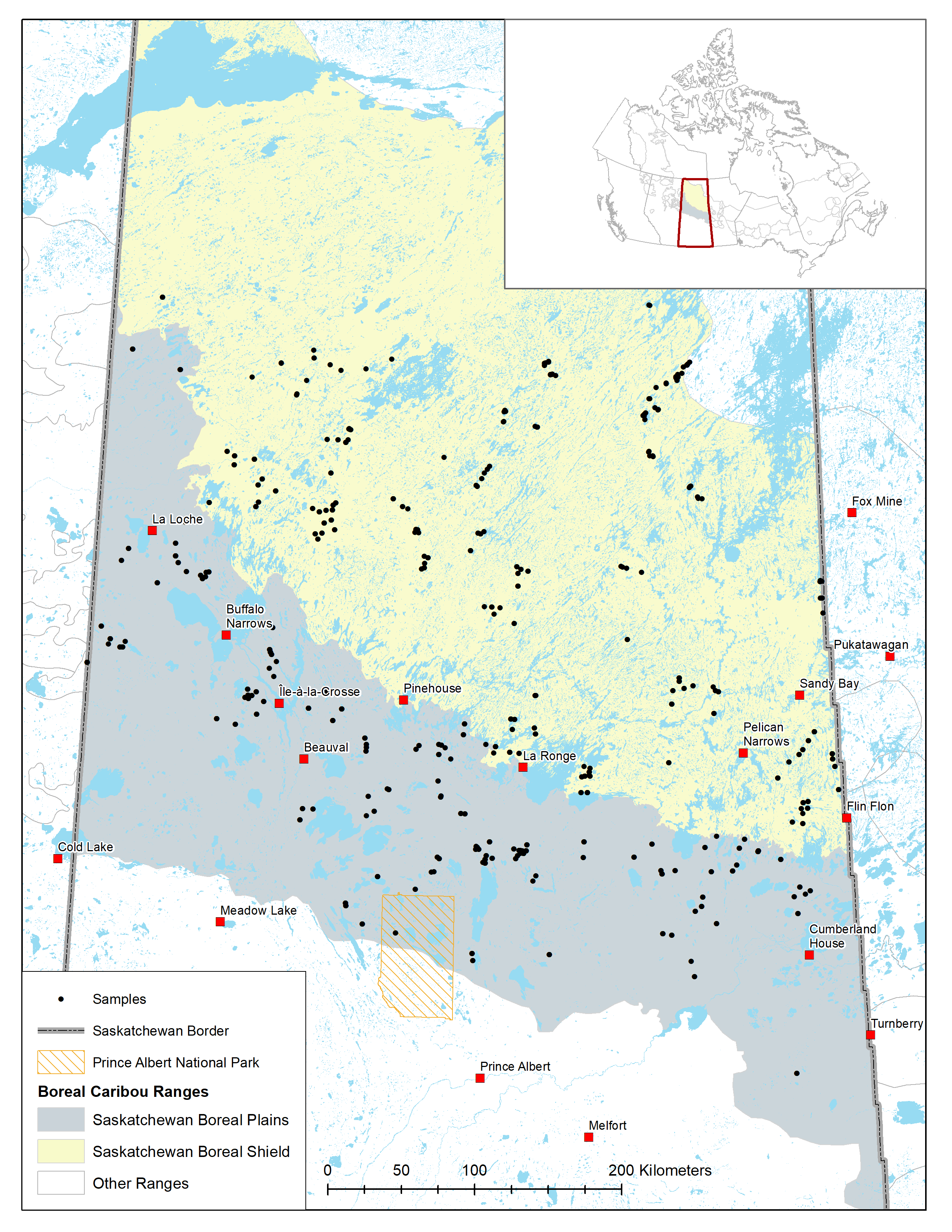


Figure S1.1: Study area and sampling surveys of boreal caribou in Saskatchewan, Canada.


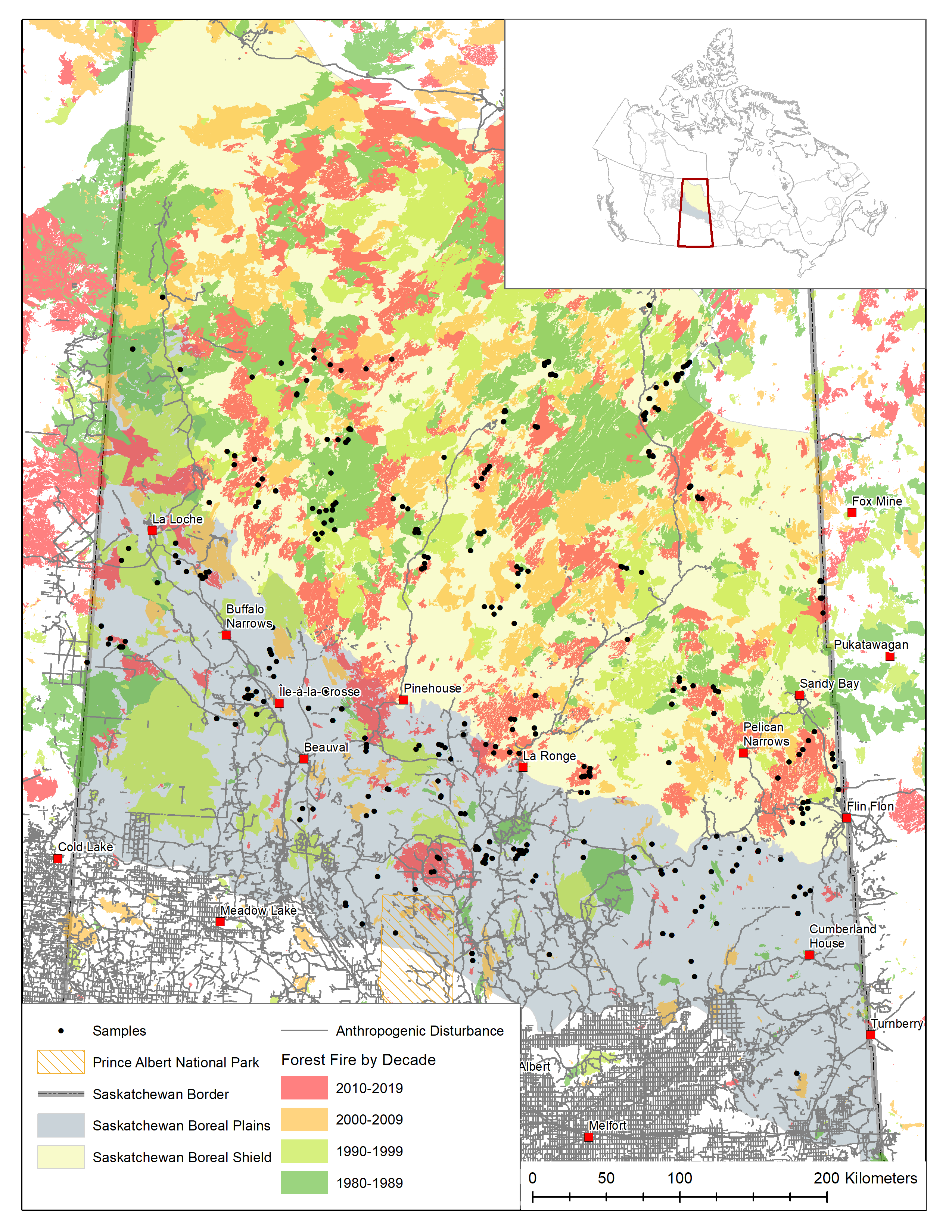


Figure S1.2: Anthropogenic and fire disturbance in Saskatchewan, Canada.

Table S1.1: Disturbance levels in the Saskatchewan Boreal Plains and Boreal Shield.

|  | Area (km²) | Fire (km²) | Fire (%) | Anthropogenic (km²) | Anthropogenic (%) | Total Disturbed (km²) | Total Disturbed (%) |
| --- | --- | --- | --- | --- | --- | --- | --- |
| Saskatchewan Boreal Plains | 103,696 | 30,411 | 29.3% | 21,122 | 20.4% | 47,903 | 46.2% |
| Saskatchewan Boreal Shield | 175,511 | 99,187 | 56.5% | 5,603 | 3.2% | 107,710 | 61.4% |

## Appendix 2: subnetworks

### Tables

Table S2.1: Ratio of edges (parent-offspring relationships) and nodes (individuals) within areas of the spatial pedigree network in Saskatchewan.

| Area | Ecozone | Nodes | Edges | Average Alpha | Average Betweenness | Average Closeness | Average Eccentricity | Average Degree |
| --- | --- | --- | --- | --- | --- | --- | --- | --- |
| Canoe Lake | Plains | 41 | 15 | 3.83 | 0.93 | 88.07 | 43.88 | 2.37 |
| SK2West | Plains | 35 | 12 | 3.74 | 0.80 | 96.30 | 45.91 | 2.34 |
| Trade Lake | Shield | 31 | 9 | 3.65 | 0.97 | 89.30 | 39.77 | 2.35 |
| Peter Pond Lake | Plains | 42 | 12 | 3.81 | 0.67 | 92.56 | 45.55 | 2.33 |
| Montreal Lake | Plains | 50 | 13 | 3.72 | 0.96 | 83.56 | 43.20 | 2.32 |
| Meeyomoot Lake | Plains | 44 | 10 | 3.59 | 0.86 | 91.13 | 48.45 | 2.30 |
| Besnard Lake | Plains/Shield | 55 | 12 | 3.73 | 0.69 | 81.99 | 46.56 | 2.24 |
| Deschambault Lake | Plains | 35 | 6 | 3.40 | 0.51 | 79.05 | 44.83 | 2.20 |
| Lac La Ronge | Plains | 35 | 6 | 3.46 | 0.34 | 83.43 | 47.97 | 2.17 |
| North PAGE | Plains | 25 | 4 | 3.40 | 0.32 | 78.81 | 43.92 | 2.16 |
| Flin Flon | Shield | 65 | 10 | 3.34 | 0.89 | 78.57 | 49.95 | 2.20 |
| Turnor Lake | Shield | 39 | 6 | 3.46 | 0.62 | 88.45 | 45.77 | 2.23 |
| Lac la Plonge | Plains | 45 | 5 | 3.22 | 0.22 | 77.28 | 46.60 | 2.11 |
| Big Sandy Lake/SK2 East | Plains | 41 | 4 | 3.39 | 0.20 | 85.78 | 43.59 | 2.10 |
| Black Birch Lake | Shield | 50 | 4 | 3.24 | 0.24 | 82.20 | 44.78 | 2.08 |
| Reindeer Lake | Shield | 38 | 3 | 3.32 | 0.16 | 80.36 | 48.71 | 2.08 |
| Cree Lake | Shield | 26 | 1 | 3.15 | 0.08 | 76.51 | 47.15 | 2.04 |
| Central SK Shield | Shield | 40 | 0 | 3.00 | 0.05 | 71.58 | 48.40 | 2.02 |

### Figures


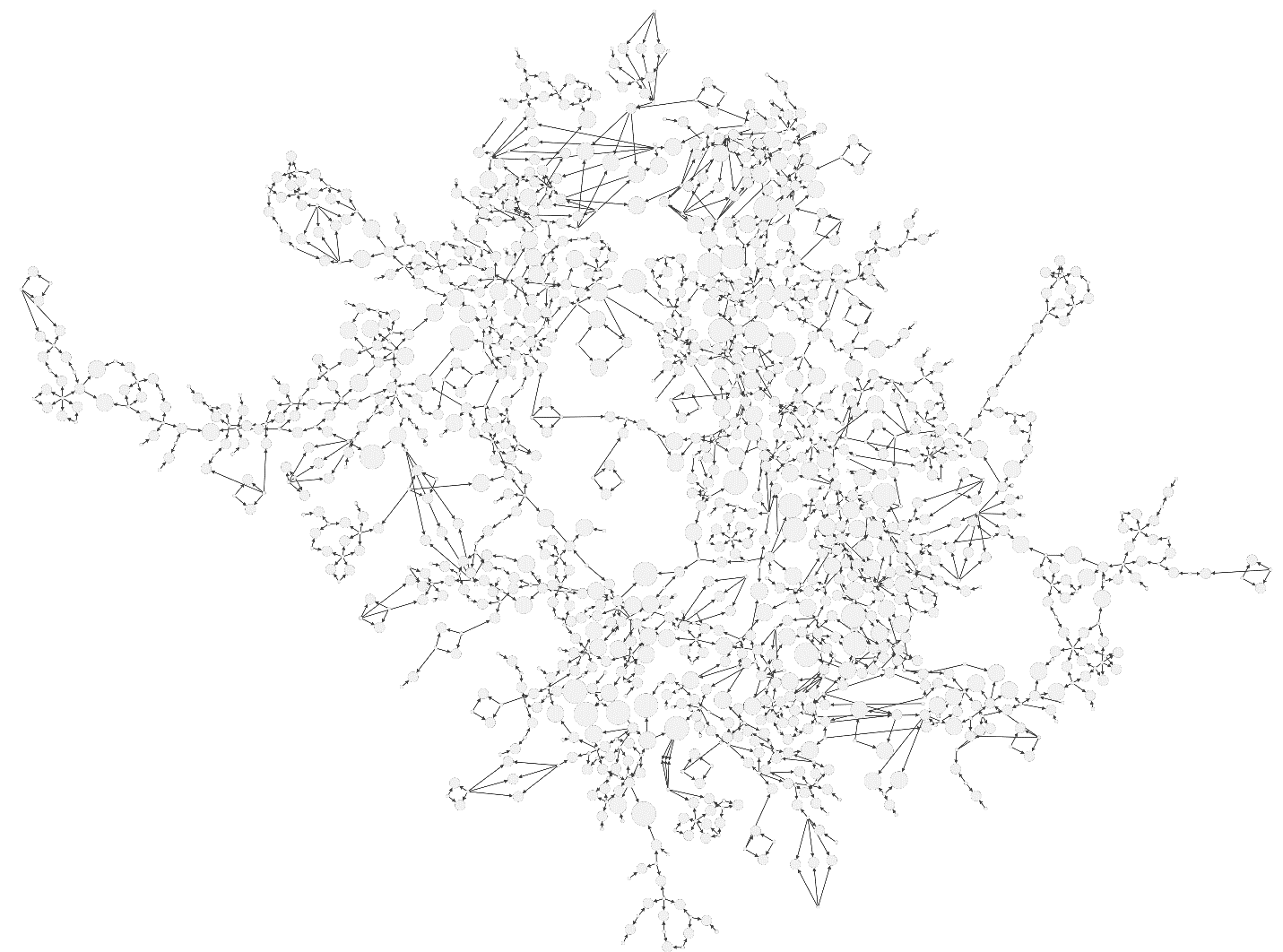


Figure S2.1: Boreal caribou full familial network in Saskatchewan, Canada. Node size indicates alpha centrality score. Edges represent parent-offspring relationships.


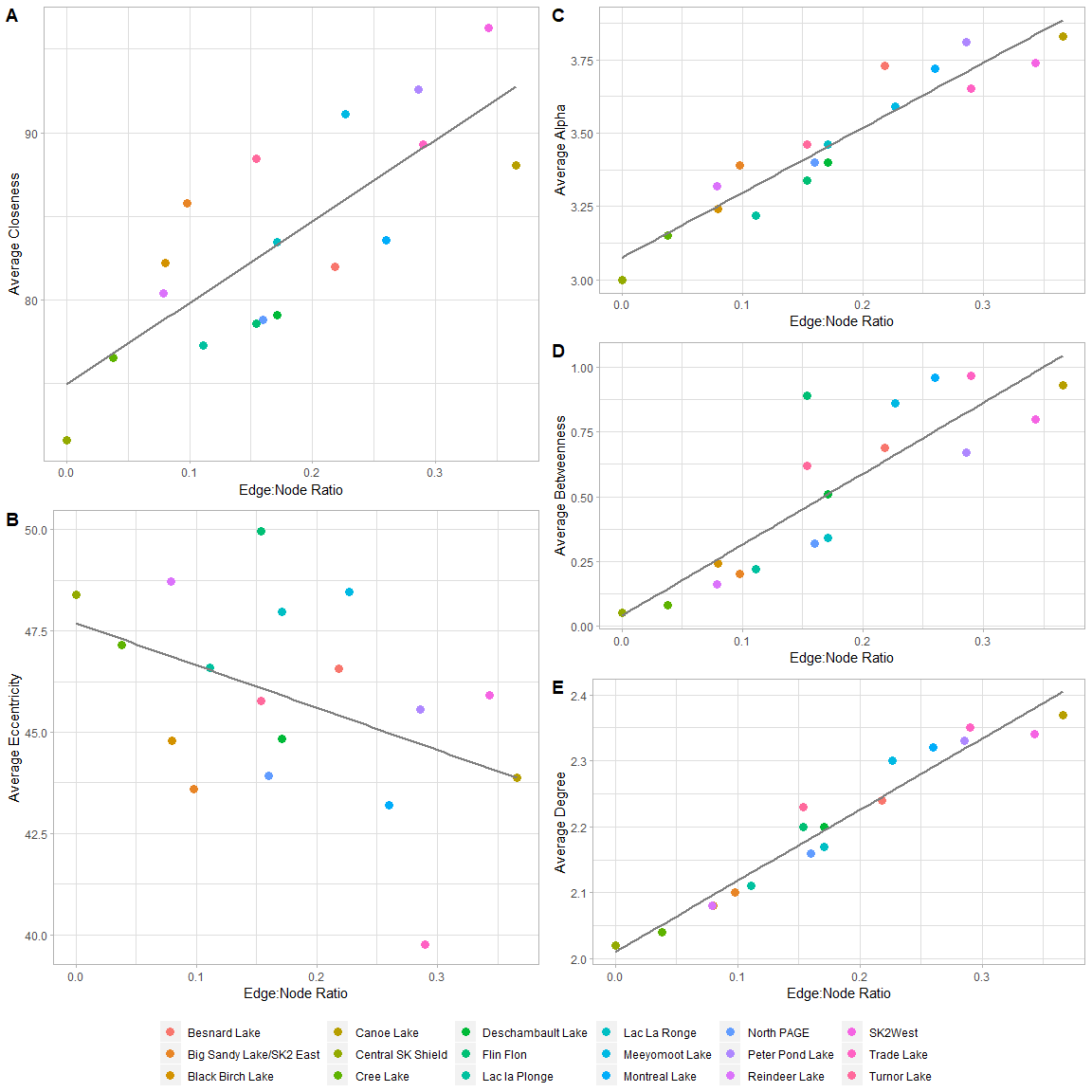


Figure S2.2: Average centrality measures with different edge:node ratios of subnetworks for the five centrality measures - closeness centrality (A), eccentricity centrality (B), alpha centrality (C), betweenness centrality (D), and degree centrality (E). Colours represent different local areas.


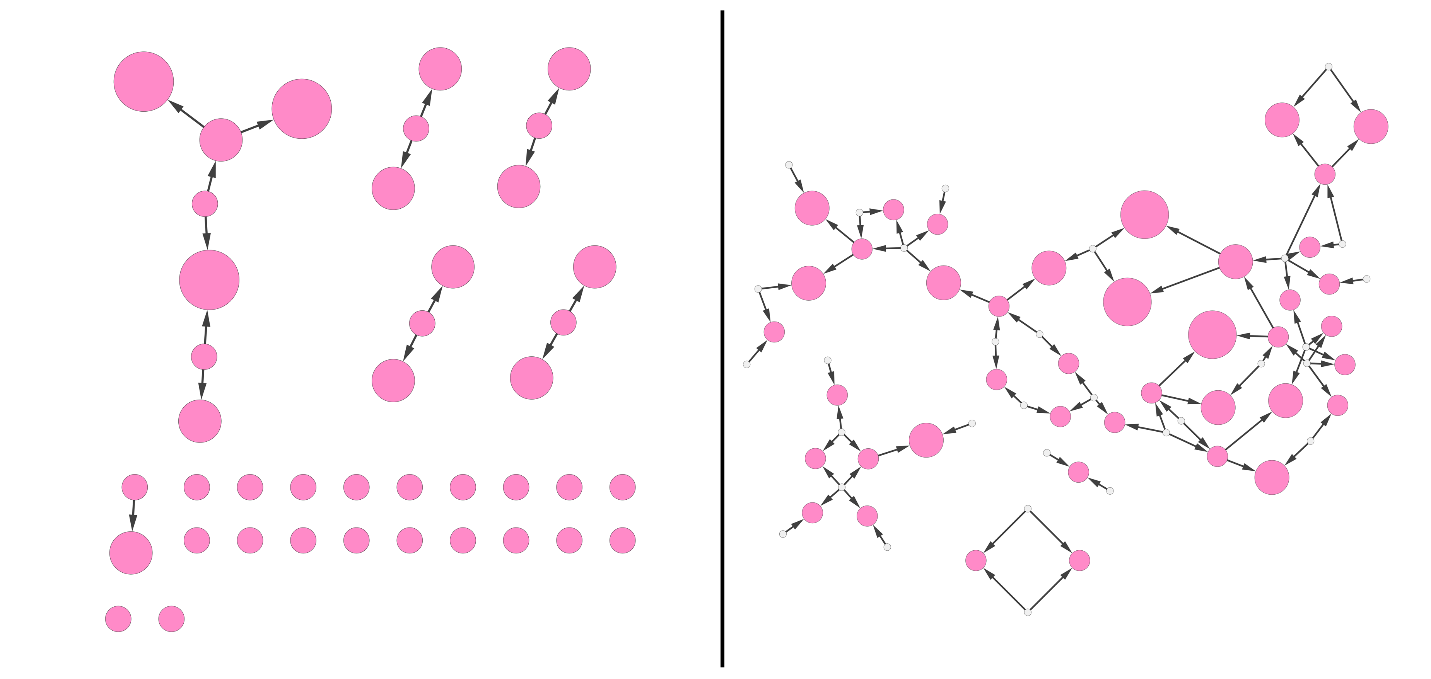


Figure S2.3: Canoe Lake high edge-to-node ratio local area network of sampled individuals (left) and with first neighbours (right). Pink nodes represent Canoe Lake individuals.


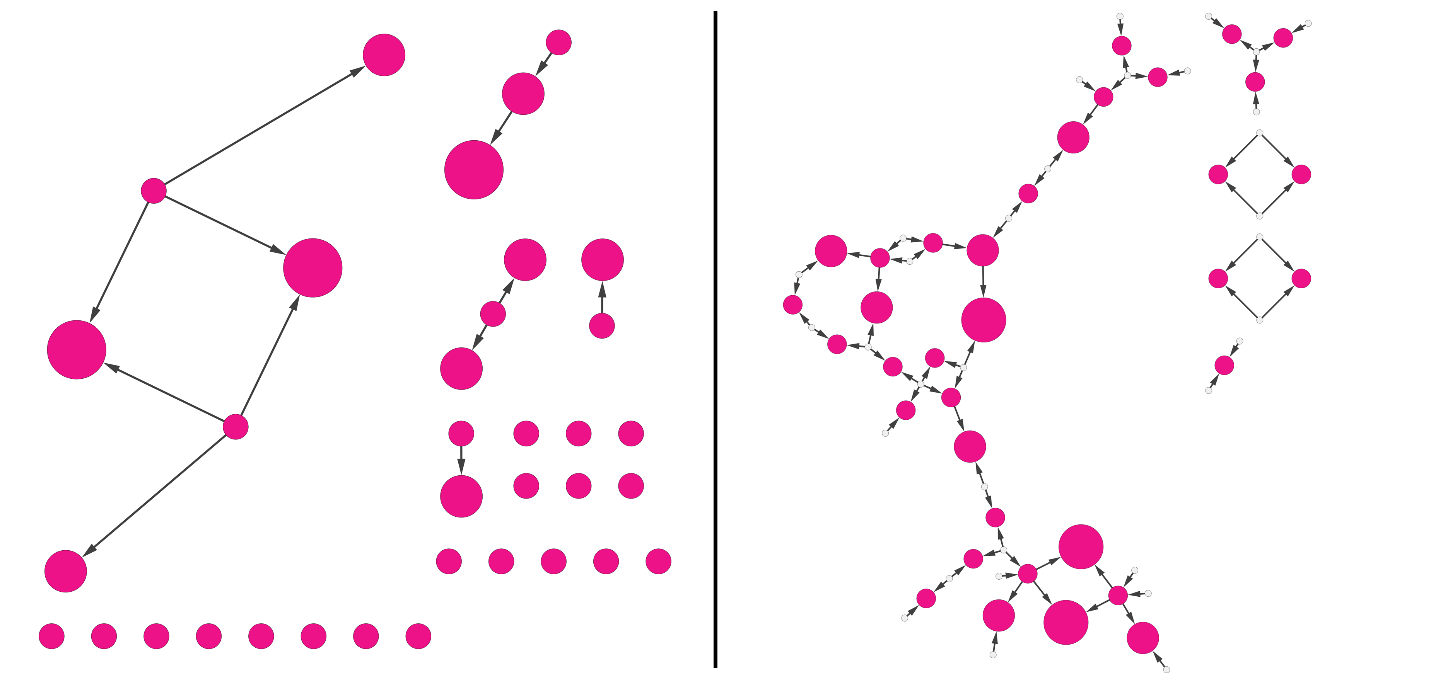


Figure S2.4: SK2West high edge-to-node ratio local area network of sampled individuals (left) and with first neighbours (right). Pink nodes represent SK2West individuals.


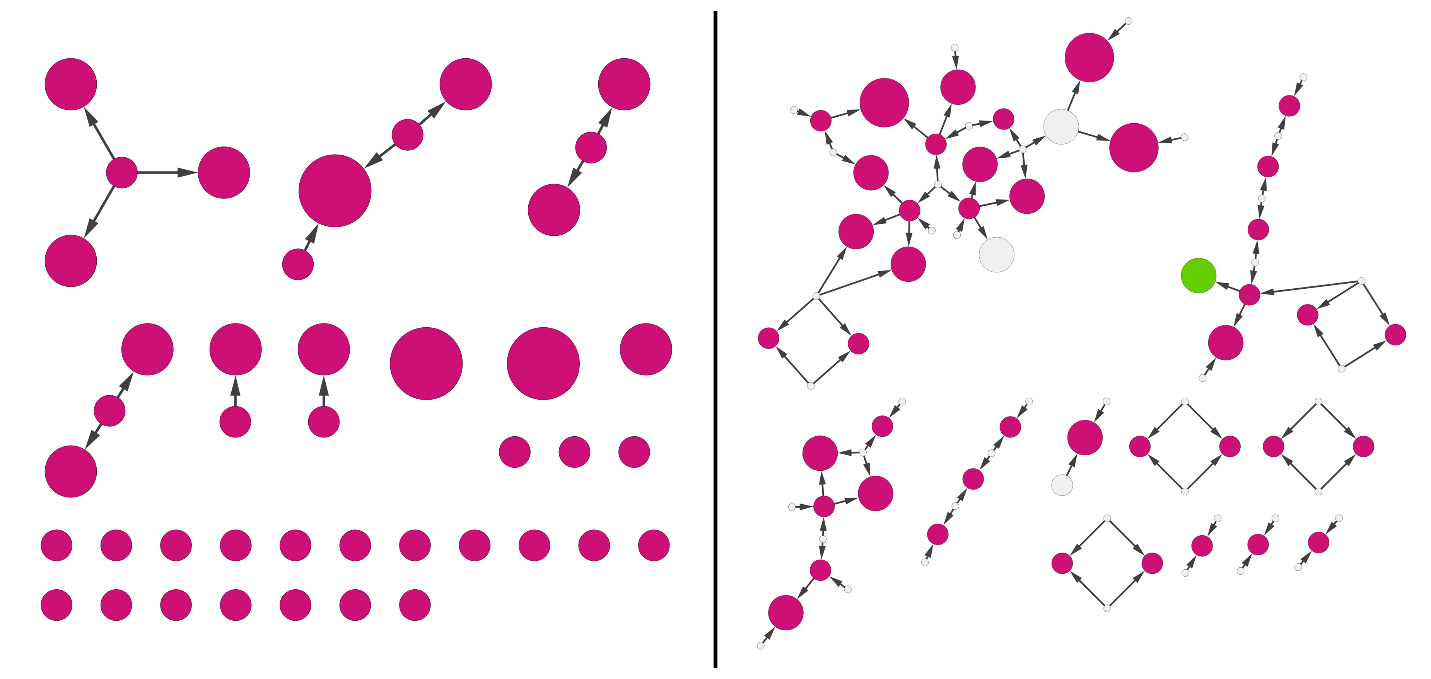


Figure S2.5: Peter Pond Lake high edge-to-node ratio local area network of sampled individuals (left) and with first neighbours (right). Pink nodes represent Peter Pond Lake individuals, green node represents Cree Lake first neighbour.


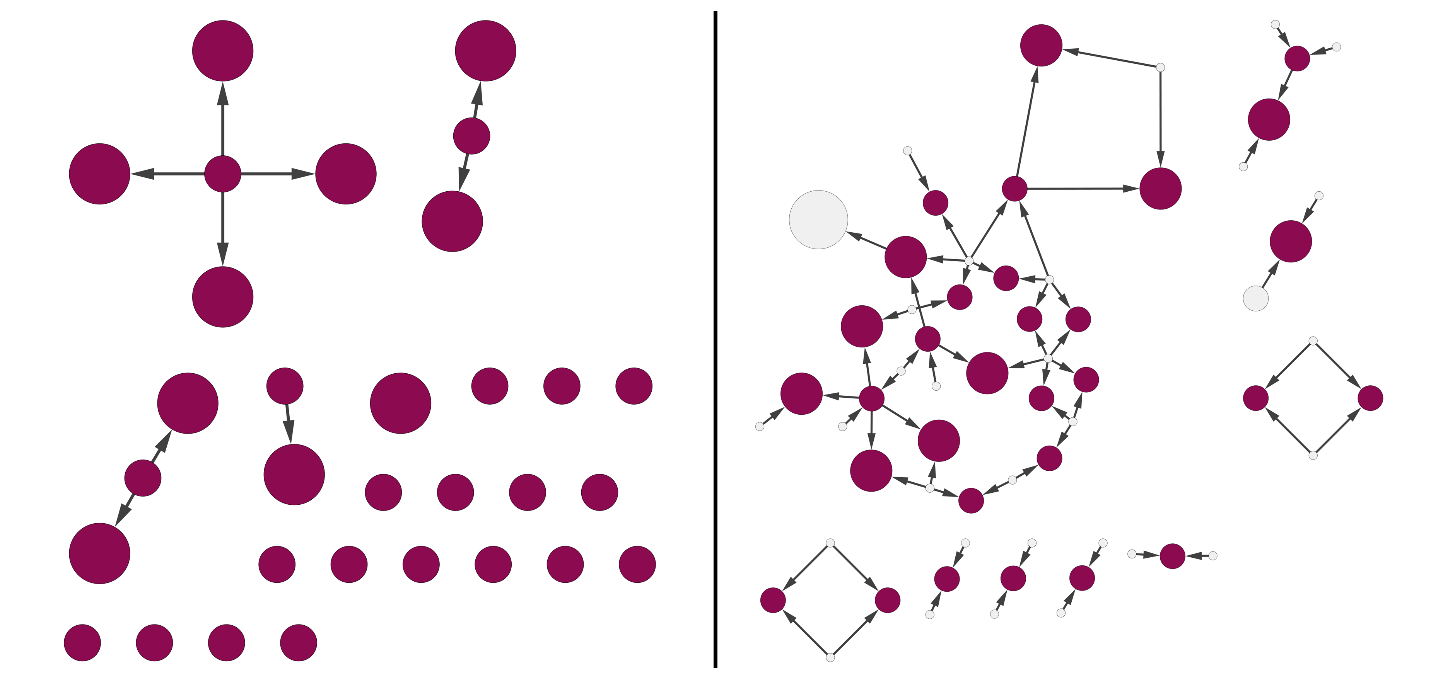


Figure S2.6: Trade Lake high edge-to-node ratio local area network of sampled individuals (left) and with first neighbours (right). Pink nodes represent Canoe Lake individuals.


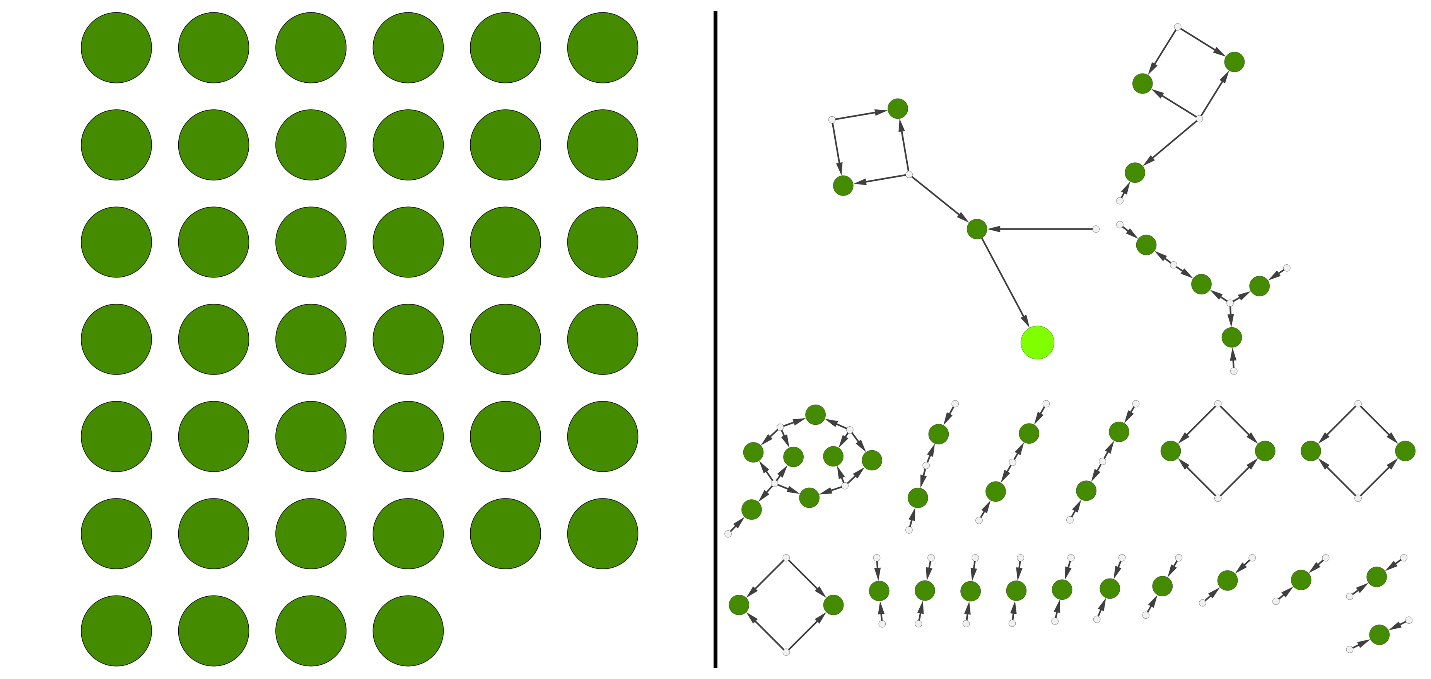


Figure S2.7: Central SK Shield low edge-to-node ratio local area network of sampled individuals (left) and with first neighbours (right). Dark green nodes represent central SK Shield individuals, light green node represents Reindeer Lake first neighbour.


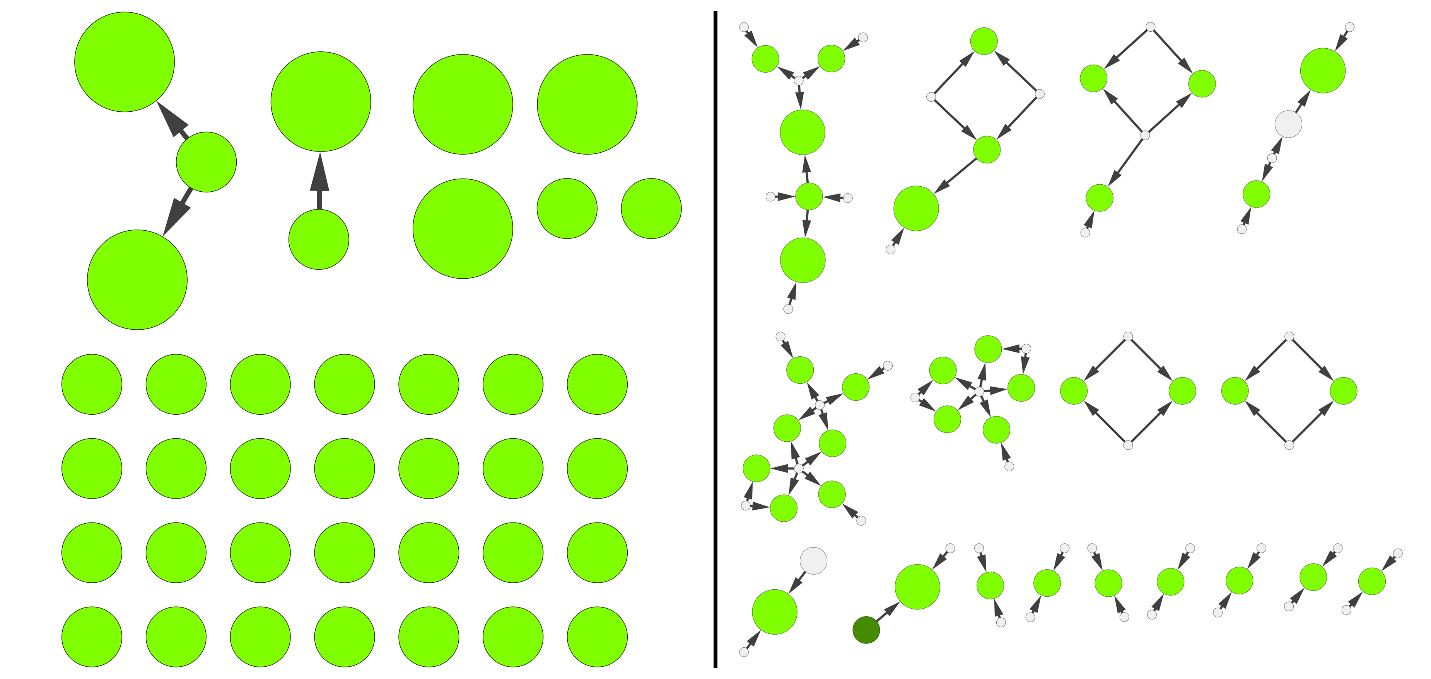


Figure S2.8: Reindeer Lake low edge-to-node ratio local area network of sampled individuals (left) and with first neighbours (right). Light green nodes represent Reindeer Lake individuals, dark green node represents Central SK Shield first neighbour.


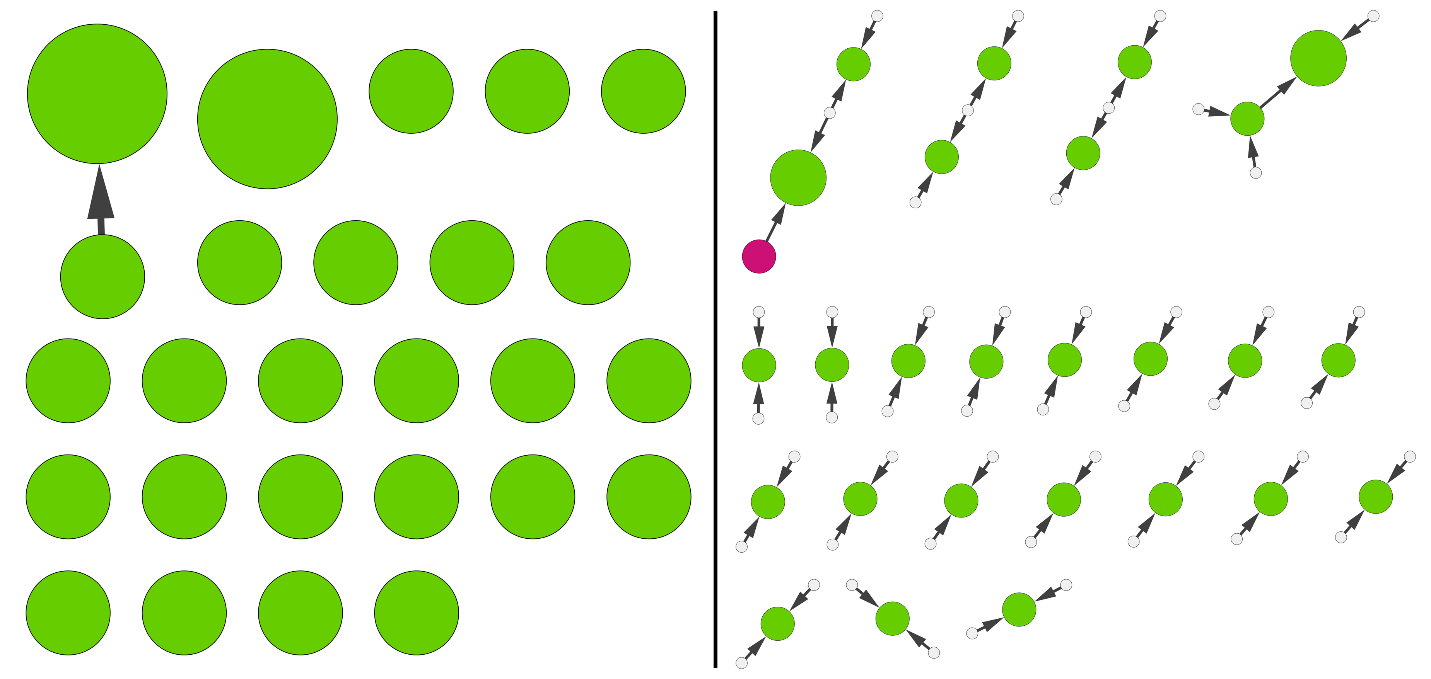


Figure S2.9: Cree Lake low edge-to-node ratio local area network of sampled individuals (left) and with first neighbours (right). Green nodes represent Canoe Lake individuals, white nodes represent inferred individuals, pink coloured node represents Peter Pond Lake first neighbour.


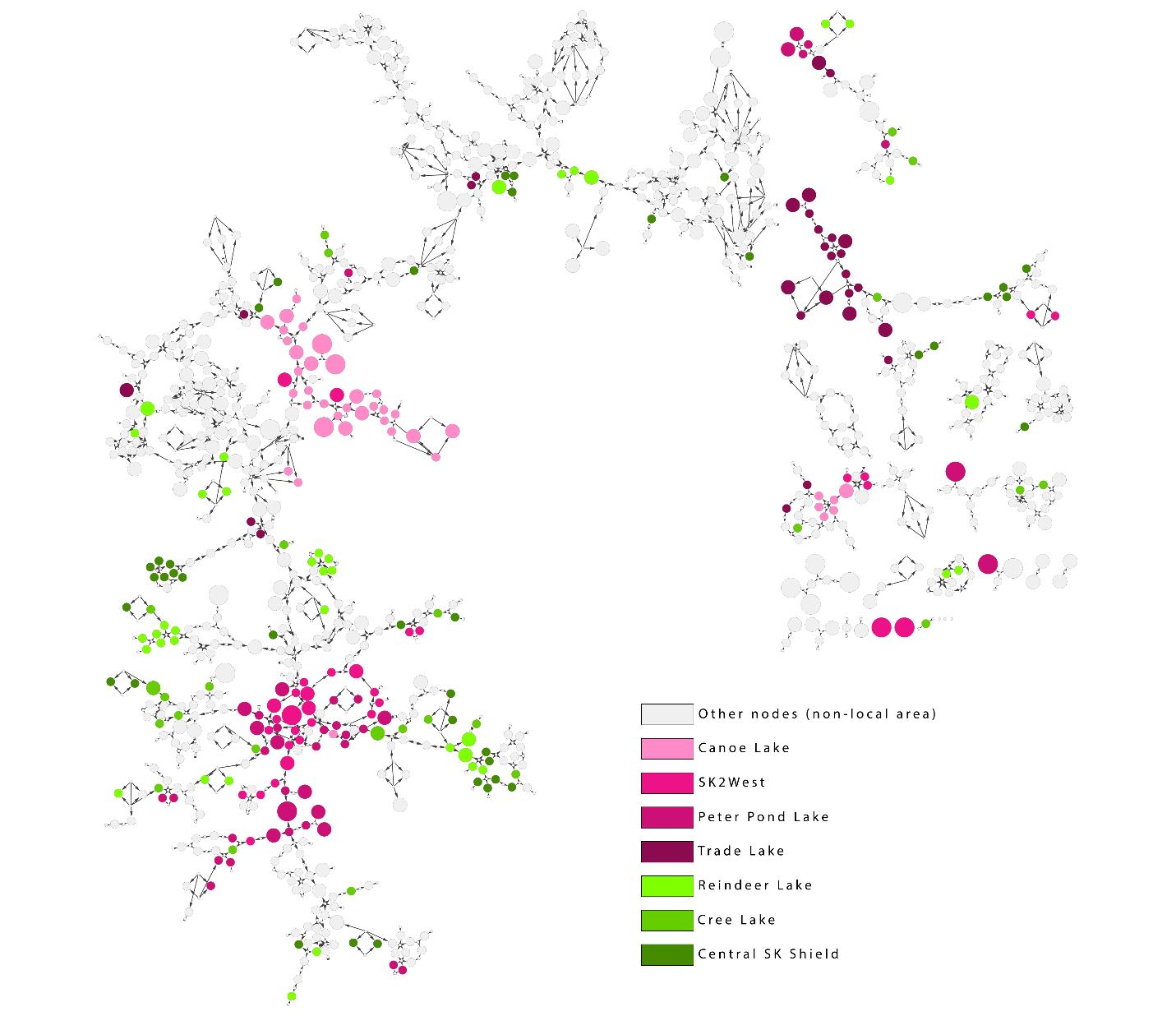


Figure S2.10: Boreal caribou familial network in Saskatchewan, Canada after removal of edges with edge betweenness > 4. Edge size indicates edge betweenness score, and node size indicates alpha centrality score. Node colour represents both local area and edge-to-node ratios. All pink nodes represent individuals from local areas with high edge-to-node ratios (Canoe Lake, SK2West, Peter Pond Lake, Trade Lake), and green nodes represent individuals from local areas with low edge-to-node ratios (Reindeer Lake, Cree Lake, Central SK Shield).
